# Community engagement in maternal and perinatal death surveillance and response: a realist review

**DOI:** 10.1186/s12884-025-08183-x

**Published:** 2025-10-14

**Authors:** Mary Mbuo, Immaculate Okello, Loveday Penn-Kekana, Brynne Gilmore, Francesca Palestra, Matthews Mathai, Merlin Willcox

**Affiliations:** 1 London School of Hygiene and Tropical Medicine, London, UK; 2 Primary Care Research Centre, University of Southampton, Aldermoor Health Centre, Aldermoor Close, Southampton, UK; 3 Education and Innovation in Health Systems (UCD IRIS), School of Nursing, Midwifery and Health Systems, UCD Centre for Interdisciplinary Research, University College Dublin, Dublin, Ireland; 4 Department of Maternal, World Health Organization, Newborn, Child and Adolescent Health and Ageing, Geneva, Switzerland

**Keywords:** Community engagement, Realist review, Maternal and perinatal death surveillance and response

## Abstract

**Background:**

Community engagement in maternal and perinatal death surveillance and response (MPDSR) could support health systems in providing people-centred care and ensure accountability for the prevention of maternal and perinatal deaths. Although community engagement activities in MPDSR have been described, the literature does not adequately explain which community engagement in MPDSR strategies succeed, the contexts in which they work, the outcomes they produce, and for whom.

**Methods:**

We conducted a realist review, which involved the identification and refinement of programme theories. An initial literature search identified four initial programme theories (IPTs) that explain how community engagement works in the different parts of the MPDSR cycle.

Six databases (Medline, Embase, Scopus, Global Health, CINAHL Plus and Web of Science) and Google were searched for papers and grey literature published between 2004 and August 2022. We used retroductive analysis on included articles to support the identification of generative causation using the heuristic of ‘context-mechanism-outcome configuration’ (CMOCs), which explained what mechanisms were triggered in different contexts and the outcomes that were produced. The findings were then used to refine the IPTs and produce final programme theories.

**Results:**

Forty-five articles from 40 studies reported some form of community engagement in MPDSR. We identified 20 CMO configurations that were synthesised into five programme theories:

1. Fear of blame demotivates community members and health professionals from engaging in MPDSR.
2. Dialogue between health professionals and community members improves collaboration and empowers community members to propose innovative solutions.
3. Trusted social connections between bereaved families and community volunteers enables them to identify and report deaths.
4. Financial and non-financial incentives motivate community members and health professionals to engage in MPDSR.
5. Community engagement is more sustainable when it is routinised and integrated into the health system.

**Conclusion:**

Implementing community engagement in MPDSR requires a systems approach that addresses the five Programme Theories collectively, rather than implementing community engagement in specific parts of the MPDSR cycle as our initial programme theories had suggested. Establishing conducive participatory spaces that promote dialogue, trust and minimise blame culture is critical for the success of community engagement in MPDSR programmes. Community members can be engaged in MPDSR processes in health facilities and community settings and high- and low-income countries.

**Supplementary Information:**

The online version contains supplementary material available at 10.1186/s12884-025-08183-x.

**Table:** 

1. What was known?
Importance of this specific problem: Implementation of maternal and perinatal death surveillance and response (MPDSR) interventions has not adequately examined the ‘mechanisms’ that support community engagement and contribute to prevention of maternal deaths Key gap to address/aim of this paper: The community engagement in MPDSR strategies that succeed, the contexts in which they work, the outcomes they produce, and for whom
2. What was done?
High-level method: Realist review Novel approach or analyses: We produced context-mechanism-outcome configurations (CMOCs) that were used to develop theories on factors that facilitate or limit community engagement in MPDSR
3. What was found specific to strengthening MPDSR implementation & action?
1. Fear of blame motivates communities and health workers to disengage from MPDSR 2. Creating conducive spaces for dialogue, such as separate community and health worker meetings, can enable more confidential discussions, minimise power hierarchies, and facilitate the exchange of different forms of knowledge during death review meetings. This can improve the quality of review sessions and increase the likelihood that community members support the implementation of recommendations 3. Working with community informants (and other intermediaries) who have strong social bonds with community members creates trusted communication channels between community members and health workers 4. Programmes can leverage on financial and non-financial incentives to motivate health professionals and community members in their engagement process. Resources for training, supervision and implementation of recommendations are essential for enabling meaningful community engagement. Providing symbolic resources such as encouragement, recognition, and value of the contributions of health professionals and community members provides intrinsic motivation for health professionals and community members 5. Embedding community involvement in routine MPDSR processes is important for sustainability
4. What are the implications for strengthening MPSDR implementation & action?
Action in programmes and/or future research gaps: future research gaps: Political Economic Analysis should be used as part of MPDSR implementation to map out how to advocate for resource mobilisation to support response

## Background

The most recent (2023) estimates for maternal and neonatal mortality and stillbirths show that progress in reducing these deaths has stagnated [1, 2]. To meet the sustainable development goal (SDG) 3 and Every Newborn Action Plan (ENAP) targets of reducing global maternal and perinatal mortality rates, we need to understand where and why these deaths happen and respond to prevent future deaths. Maternal and Perinatal Death Surveillance and Response (MPDSR) could support efforts to accelerate progress in the prevention of deaths in health facilities and in the community [3, 4]. MPDSR is seen as an accountability process that can link data to response, improve quality of care and mobilise resources for maternal and newborn health [5, 6].

**Fig. 1:**
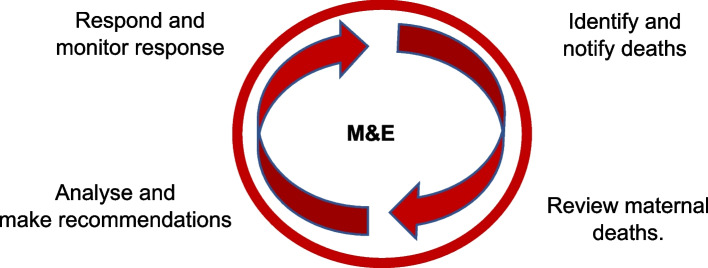
MDSR cycle [8]

The primary goal of MPDSR is to prevent avoidable maternal and perinatal deaths by systematically collecting, analysing and aggregating information on maternal and perinatal deaths to guide decision-making [4]. MPDSR builds on existing strategies for reviewing maternal and perinatal deaths in health facilities and communities which are confidential enquiries, maternal and/or perinatal death reviews, verbal and/or social autopsies and community-based reviews [4, 7–9]*.* Regardless of the strategies used to review deaths, the ultimate purpose of the MPDSR process is to complete the action cycle by linking surveillance data to response [7, 10, 11] See Fig. 1.

MPDSR is a complex process that involves different activities taking place at different levels of the health system [7]. Primary care facilities conduct death reviews and develop action plans to address modifiable factors within the facility and in the communities that they serve [7, 12]. The national and sub-national levels of the health system rely on the information generated at primary care facilities during death review meetings to prioritise their actions [7]. At the national level, the MPDSR process is expected to collate data (from death reviews) from sub-national levels to guide national level priority setting [7, 13]. The MPDSR process brings together different stakeholders such as health professionals, civil society, private sector, professional associations (such as nursing/midwifery councils) and community members to work together to support review and response efforts [4, 5, 14–17].

Community engagement is a process through which community members, health professionals and other stakeholders build relationships and work together to improve the health and well-being of individuals and communities [18]. In the context of MPDSR, community members can include people with shared geography, bereaved family members, community health workers or volunteers, community representatives (such as village elders or religious leaders) and civil society organisations (CSOs) [19]. Community members are key to MPDSR processes because they can provide critical information about social factors and quality of care issues that contribute to deaths [4–6].

There is some evidence that community members can participate in different parts of the MPDSR cycle [7, 20–22]. They can support active surveillance by identifying maternal and perinatal deaths, not only in the community, but also in health facilities (especially those which do not actively participate in MPDSR, like private and informal facilities) [10, 22–26]. They can also participate in community and facility death review meetings and support the implementation of recommendations [21, 27–29]. Table 1 shows examples from the literature of community engagement activities in different parts of the MPDSR cycle.

**Table 1:** Community engagement in different parts of the MPDSR cycle [30]

Steps in MPDSR cycle	How community members are engaged
Identification and notification of deaths	Identify and notify all maternal and perinatal deaths occurring in the community and provide the information to health workers
Review of maternal/perinatal deaths and identification of actions to address modifiable factors identified in the review process	Community members provide information to facilitate classifications for assigning cause of death through verbal autopsy Community members involved in verbal and social autopsy sessions to discuss maternal/perinatal contributors of specific deaths in the community Community members participate in death reviews at health facilities and provide information on social factors prior to arriving at a health facility and experiences of care within health facilities to MPDSR committees Community members involved in verbal and social autopsy sessions to propose community -level actions to address modifiable or avoidable factors identified during the review process
Response	Community involvement in implementing community-level actions to prevent maternal/perinatal deaths
Monitor and evaluate	Community members involvement in advocacy with duty bearers/health workers and policy makers to support implementation of recommended actions

There have been several literature reviews on MPDSR including a scoping review of implementation factors in low and middle income countries (LMICs) [7], a systematic review on qualitative studies on MPDSR which focused on the functionality of the MPDSR process in LMICs [31] and implementation of MPDSR in humanitarian settings [32] among others. While these literature reviews have been useful in unpacking implementation of MPDSR, none of the reviews have specifically focused on community engagement. Policy guidelines and researchers agree that community engagement can support the MPDSR process to achieve its overall goal of preventing maternal and perinatal deaths [5, 6, 8], but the existing research and guidelines do not offer specific advice on how to do this in practice.

We conducted a realist review [33] to develop and refine theory to answer the question: what community engagement strategies support MPDSR, in which contexts, what outcomes do they produce and for whom? Given that both community engagement and MPDSR are complex processes [7, 34], a realist review is best suited to explain how community engagement in MPDSR works in different contexts and the underlying mechanisms that trigger outcomes [35, 36].

## Methods

Realist review or synthesis is a theory-driven approach that examines the underlying assumptions of how an intervention works, (or does not work), thus providing pathways to improve implementation [35, 37, 38]. Realist reviews go beyond examining whether interventions work, by explaining why they result in certain outcomes [39]. By using a realist review approach, we aim to investigate the underlying assumptions of community engagement in MPDSR programmes and illustrate how different contexts influence the outcomes. We used a realist review to identify the resources introduced to MPDSR processes (for example, providing funds for health workers to supervise community volunteers) and the reasoning behind providing these resources (for example, supervising community volunteers creates opportunities for health workers to listen to concerns from community volunteers). We then examined the links between the resources introduced and the reasoning behind the decisions [39] that programme implementers used to develop programme theories on the reasons why community engagement in MPDSR succeeds or fails to succeed to produce programme theories.

This realist review was conducted in two stages between May 2022 and March 2023. A complete description of the process has been published separately and the study protocol published on Prospero (ID: CRD42022345216 [19]. During the first stage, we used the findings of an unpublished literature review [40] to develop our research question and initial programme theories on how community engagement in MPDSR is expected to work. We presented the research question and the rationale for the realist review to the WHO MPDSR Technical Working Group (WHO-MPDSR-TWG) subgroup on community engagement and blame culture. Community engagement in MPDSR was part of the 2020–2023 workplan of the global TWG and through this forum, the members gave feedback on the framing of the research question and initial programme theories (IPTs) as part of implementation of the workplan. Through a consultative process with the TWG, we identified four initial programme theories. These are:

(i) Community engagement supports data collection and facilitates the reporting of maternal and perinatal deaths occurring in the community corresponding with the identification, notification, verbal autopsy, and review steps of the MPDSR cycle.
(ii) Community engagement supports improvement in quality of care by providing opportunities for community members to share their experiences of care before a maternal and/or perinatal death. Health professionals can engage community members in MPDSR by conducting verbal and social autopsies, and community-based reviews. These can empower community members to give feedback on issues such as disrespectful maternity care. If quality of care is improved as a result, care seeking should also improve.
(iii) Community members can participate by making recommendations and implementing local-level solutions to address some of the material and social barriers identified as contributors to maternal or perinatal deaths, e.g. supporting transport arrangements for pregnant women to facilitate timely childbirth.
(iv) Community members can be involved in advocacy with duty bearers/health providers and policymakers to support the implementation of recommendations determined through the MPDSR process.

This paper is aligned with the Realist And Meta narrative Evidence Syntheses: Evolving Standards (RAMESES) training materials and publication standards [35]. Some of the co-authors (MMb, IO and MMa) of this realist review are from LMICs and all co-authors involved in this review have experience working on MPDSR in LMICs. Similarly, all the members of the advisory team for this review come from LMICs and have experience implementing MPDSR programmes in LMICs.

### Search strategy, inclusion/exclusion criteria

During the second stage, a systematic search of the literature was conducted to identify any relevant resources that could support IPT theory refinement. Three broad concepts and their variations were used for the search- community engagement’, ‘maternal and perinatal death’, and ‘surveillance and response.

Table 2 provides a comprehensive list of the search terms used in this realist review.

**Table 2:** Search terms community engagement in MPDSR

Community Engagement terms	“Collective or community or community intervention” or “community action” or “community mobilisation” or “capacity building” or collaboration or conscientization or engagement or intervention or outreach or involvement or consultation or “shared leadership” or “community network” or “community participation” or leadership or “health program” or “community initiative” Empower* or “Health Promotion” or “Maximi? ing access” or “Participatory intervention” or “Participatory approach” or “Social mobilization” or “Social movement” or “Social capital” or “Social participation” or “Village health worker” or “Women group” or “community capability” or “collective efficacy” or “patient public involvement” or PPI or “patient public engagement” “Consumer participation” or engagement or involvement or “community representation” or “community accountability” or “community W3 accountability” or representation or “social accountability” or “community advocacy” or “community health worker” or “community representative” or “health facility committee” or “health management committee” or “Stakeholder participation” or “stakeholder engagement” or “health co-production”
Maternal or Perinatal death	“Maternal death” OR “mother death” OR maternity OR fetal OR perinatal OR pregnancy OR "child-birth" OR birth OR "labo?r W/3 mortality" OR death* OR fatality* OR “pregnancy complication” OR “f?etal death” OR “still-birth” OR “still-born” OR “sudden infant death” OR sids OR “cot death” OR “crib death” or “saving mothers lives” OR “making pregnancy safer” OR “making childbirth safer” OR “new-born death” OR “intrapartum death” OR “intrapartum mortality”
Surveillance and Response	"maternal and perinatal death surveillance and response" or MPDSR or “maternal death surveillance and response” or MDSR or audit or surveillance or response or "death audit" or “maternal death review” or perinatal death review” or "death surveillance" or "death review" or "surveillance W3response" or "confidential enquir*" or "confidential inquir*" or "death* meeting" or "death enquir*" or "death inquir*" or "confidential enquir* into Maternal and Child Health" or CEMACH or "Confidential Inquir* into Maternal and Child Health" or CIMACH or "Cent* for Maternal and Child Enquir*" or CMACE or "Cent* for Maternal and Child Inquir*" or CMACI or "Confidential Enquir* into Maternal Death" or CEMD or "Confidential Inquir* into Maternal Death" or CIMD or "Cent* for Maternal Death Enquir*" or CMDE or "Cent* for Maternal Death Inquir*" or CMDI or "verbal autops*" or "social autops*" or "communit* W3 death audit" or "death review" or "death meeting" or "verbal autops*" or "social autopsy"

The literature search for this realist review is limited to papers published from 2004 to coincide with the publication of the first WHO maternal death review guideline [41]. The search covered countries from all income levels and included articles in any language; however, search terms were only in English. This realist review included published papers and grey literature that could contribute to theory building or testing of community engagement in MPDSR. We also included commentaries or opinion pieces emphasising the need for community engagement in MPDSR.

Papers were included if they reported community engagement in any of the components of MPDSR, i.e., death surveillance, verbal and social autopsy, confidential enquiry and death review meetings for either maternal and/or perinatal deaths, even if the action cycle was not closed. For instance, we included articles that described community involvement in collecting information about deaths or conducting verbal autopsies, even if the articles did not report on the review or response aspects of the process. However, at the full screening stage, we excluded articles that did not report on community engagement in sufficient details to allow for theory development or refinement. See Table 3 for inclusion and exclusion criteria.

**Table 3:** Inclusion and exclusion criteria

Component	Inclusion criteria	Exclusion criteria
Search terms	Community engagement in death surveillance, verbal and social autopsy, confidential enquiries and death review meetings for either maternal and/or perinatal deaths	Maternal or perinatal death reviews with no community engagement component reported
Publication type	Published and grey literature including editorials and commentaries and NGO reports	None
Study type	Qualitative, quantitative, and mixed methods	None
Date	2004 to August 2022	Any date before 2004
Language	All languages (search conducted in English)	No language barriers
Countries	All income levels	None
Appraisal for relevance and richness. As Dada et al. [42] note, the relevance and richness of papers for theory development/refinement should be part of the inclusion criteria to ensure that only studies that contribute to theory refinement or testing are included	Papers that provide rich descriptions of how community members are engaged in MPDSR to contribute to theory development or refinement	Papers that do not provide adequate details on how community members are engaged in MPDSR

Six databases (Medline, Embase, Scopus, Global Health, CINAHL Plus and Web of Science) were searched. Members of the WHO MPDSR Technical Working Group and non-governmental organisations (NGOs) implementing MPDSR projects were contacted to identify additional published and grey literature on community engagement in MPDSR. A Google search using the key search terms to identify additional published and grey literature was also conducted. In addition, we hand-searched reference lists to identify any other resources and contacted authors for additional information. Cluster searching was used to identify sibling studies (articles from the same study) [37]. By including sibling studies, we gained additional insights on the contexts or underlying assumptions of how interventions are expected to work [37].

### Study selection

Titles and abstracts were uploaded onto Eppi-Reviewer 4 (https://eppi.ioe.ac.uk/EPPIReviewer-Web/home) and were screened independently by two authors (MMb and IO). Two other members of the team (LPK and AP) re-screened 20% of titles and abstracts [43]. There was a 90% consensus among the screening team regarding papers meeting inclusion criteria. Disagreements were discussed and resolved by MMa, a co-author who is an expert on MPDSR. Two team members (MMb and IO) screened full texts to identify papers that reported community engagement in MPDSR in sufficient detail to allow for analysis.

### Appraisal

Articles were appraised in detail for relevance, richness and rigour [35, 42]. Relevance was used to ensure that they described some form of community engagement in any part of the MPDSR cycle, and richness to ensure there was sufficient detail about how an intervention works and/or details of the context in which the intervention is implemented to support theory development [44]. We used quality assessment tools to appraise articles for rigour (CASP for peer-reviewed articles and AACODS for grey literature) [45, 46].

Papers were included if they provided thick descriptions that could contribute to theory refinement, even if the description of the methods was not sufficiently robust [39]. Articles were grouped into three categories for relevance and richness- as ‘high’, ‘moderate’ or ‘low’ [42], based on the level of detail that they provided on how community engagement is expected to work in any part of the MPDSR cycle. Articles rated as high and medium in richness were included, and those rated low were excluded as they did not contain enough detail to support theory development.

### Data extraction

We created a table in MS Word to extract data from the included articles and held discussions among the co-authors to pretest the suitability of the extraction table. Two authors (MMb and IO) extracted data on article characteristics such as study location, study type, part of MPDSR cycle that community members are engaged in and any reference to theoretical frameworks on community engagement in the study.

Context-Mechanism-Outcome Configurations (CMOCs) were first extracted from articles ranked as ‘high’ in terms of richness. Retroductive analysis was used on the article’s texts to identify generative causation, i.e. how outcomes are generated within a certain context through a triggering mechanism [42]. The CMOCs were discussed among co-authors and then presented to the WHO-MPDSR-Technical Working Group for feedback in October 2022. We then extracted data from articles appraised as medium in terms of richness. This additional information either confirmed or refuted the CMOCs generated during the first stage, i.e. articles rated as ‘rich’. Finally, we extracted contextual information from sibling studies for theory refinement. We triangulated CMOCs from less methodologically robust papers with others to ensure that more than one article could support theory development or refinement [43]. The first author (MMb) corresponded with two authors of included studies to gain additional insights for theory development and refinement.

### Synthesis and refinement of programme theories

CMOCs were categorised into prominent themes or demi-regularities by examining recurrent patterns in contexts, mechanisms and outcomes [38]. We also used existing theoretical frameworks on community engagement in health to theorise how the contexts trigger different mechanisms and outcomes [47]. Through this process, we refined our initial programme theories and produced five programme theories (PTs).

## Results

**Fig. 2:**
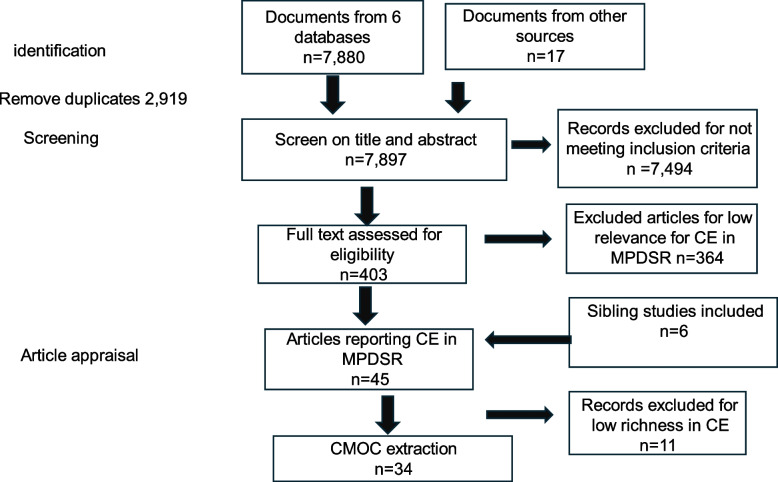
Article screening flowchart

Figure 2 shows the flow diagram of our screening process. An initial search identified 7,880 articles from the six databases and 17 articles from a Google search and NGO contacts. After removing 2,919 duplicates, we screened articles for inclusion criteria and appraised the articles for relevance and richness for community engagement in MPDSR.

### Characteristics of articles describing community engagement in MPDSR

Forty-five articles from 40 studies reported some form of community engagement in MPDSR. 40 of the 45 articles that report community engagement in surveillance and response are from LMICs, many of which were from Bangladesh [11, 14, 18, 37, 40, 48–50] and India [51–58]. We included two studies from high-income countries; one study covering six countries [59], and one study from the United Kingdom, reported in four articles [49, 50, 60, 61]. Five of the 45 articles were grey literature [53, 57, 62–64] and one was an opinion piece [65]. Details on the study characteristics can be found on Table 4

**Table 4:** Study characteristics

Author/Year	Location	Publication type		Study design	Intervention	Level of health system CE in MPDSR is reported	Part of MPDSR cycle where CE is reported
High relevance and rich description on community engagement in MPDSR							
Ayele et al. (2019) [10]	Ethiopia	Peer review		Cross-sectional Mixed methods evaluation of a pilot programme	MPDSR	Tigray region	Notification and reporting Involving Kebele chairman and Women Development leaders in MDR steering committee
Bakhbakhi et al. (2017) [60]	England	Peer review	Qualitative		Perinatal death reviews	Southwest England	Review
Bayley et al. (2015) [21]	Malawi	Peer review		Mixed method pilot study	Maternal death review	Mchinji District in Malawi	Notification and reporting Review Response
Biswas et al. (2016) [48]	Bangladesh	Peer review		Qualitative	Maternal and neonatal death reviews	Thakurgaon and Jamalpur districts of Bangladesh	Social autopsy for review and response
Burden et al. (2021) [61]	UK	Peer reviewed		Mixed methods	Perinatal death reviews	One tertiary maternity unit	Death review
Hutain et al. (2019) [20]	Sierra Leone	Peer review		RCT (mixed methods)	Verbal autopsy for child deaths (disaggregated by age and reported neonatal deaths)	Free Town, Sierra Leone	Notification and reporting Review and response
Igumbor et al. (2020) [66]	South Africa	Peer review		Mixed methods	Verbal and social autopsy for maternal and perinatal deaths	Khayelitsha,(informal settlement) Sub-district in Cape Town	Notification and reporting Community-based review
Kalter et al. (2011) [51]	India	Peer review		Mixed methods	MPDSR	India	Notification and reporting Community-based review and response
Mahato et al. (2018) [65]	Bangladesh	Peer review		Opinion piece	MPDSR	Bangladesh	Social autopsy
Moyer et al. (2016) [27]	Ghana	Peer review plus grey literature		GIS mapping and intervention at DHS surveillance sites	Social autopsies for maternal and neonatal deaths	One district in Ghana	Notification and reporting Community-based review and response
Patel et al. (2007) [52]	India	Peer review		Qualitative	Neonatal death review	India Uttar Pradesh	Notification and reporting Community-based review and response
Prata, Gerdts and Gessesse (2012) [67]	Ethiopia	Peer reviewed		Mixed methods	Pilot intervention study to assess feasibility of a community-based approach to measuring maternal mortality	Three health posts, I health centre in one region-Tigray	Notification and reporting
SAHAYOG (2016) [53]	India	Grey, published		Qualitative	Social autopsy for maternal death reviews	seven districts from the states of Odisha, West Bengal, Jharkhand and Uttar Pradesh	Monitoring and Advocacy
Studies with moderate relevance and richness for community engagement in MPDSR							
Abebe et al. (2017) [68]	Ethiopia	Peer reviewed		Qualitative	MDSR	Four zones in 4 regions (West Gojjam Zone in Amhara Region, West Arsi Zone in Oromia, Guraghe Zone in SNNPR and Southern Zone in Tigray	Notification and reporting
Adair et al. (2020) [24]	(Bangladesh, Colombia, Myanmar and Papua New Guinea	Peer reviewed		Case studies	Community death surveillance	(Bangladesh, Colombia, Myanmar and Papua New Guinea	Death notification
Anwar et al. (2018) [69]	Pakistan			Population-based study to estimate maternal and perinatal mortality	MPDSR	District of Abbottabad of Khyber Pakhtunkhwa province, located in the North of Pakistan	Notification and reporting
Barnett et al. (2008) [54]	India	Peer review		RCT	Birth and death surveillance	India – Orissa and Jharkhand	Notification and reporting
Biswas, Rahman, Halim et al. (2014) [70]	Bangladesh	Peer review		Mixed methods	Maternal and neonatal death review	Kashipur Union	Notification and reporting Social autopsy for review and response
Biswas (2017) [11]	Bangladesh	Peer review		Document review	MPDSR	National	Notification and reporting Community-based review
MCSP Tanzania (2018) [64]	Tanzania	Published Grey		Qualitative	MPDSR	National Regional Facilities from 2 regions in Tanzania	Death notification and reporting
MCSP Zimbabwe (2017) [63]	Zimbabwe	Published, grey literature		Qualitative	MPDSR	16 hospitals across six provinces in Zimbabwe	Notification and reporting
Melberg et al. (2019) [71]	Ethiopia	Peer review		Qualitative	MDSR	Addis Ababa	Reports on blame culture and how it affects notification and reporting
Mir et al. (2015) [72]	Pakistan	Peer review		Quantitative and qualitative	Maternal death surveillance	Chakwal district	Notification and reporting
Moshabela et al. (2015) [73]	Senegal	Peer review		qualitative	MDSR	Millenium Village Project	Notification and reporting
Nabukalu et al. (2019) [74]	Uganda	Peer reviewed		Cross-sectional survey	Verbal autopsy for child deaths (some of which were neonatal)	Kasese district, Western Uganda	Notification and reporting Community-based review
Options Consultancy Ltd & PHRI (2022) [62]	Nigeria	Grey literature		Project evaluation	MPDSR	Kaduna State	Notification, reporting and community-based review
Qomariyah (2010) [26]	Indonesia	Peer review		Quantitative	Maternal death surveillance	Serang and Pandeglang districts, Banten Province, as in all of Indonesia, 708 villages	Notification and reporting
Willcox et al. (2018) [29]	Mali and Uganda	Peer reviewed		Mixed methods	Child deaths (neonatal deaths included)	Mbarara district: the urban parish of Kibuli and Kayonza sub county in Uganda Kolokani district and Sikasso district in Mali	Community-based death notification and review

One abstract was in French (with an English translation), but the article focused on near miss reviews and did not provide sufficient details on community engagement in MPDSR and was excluded [75].

### Articles included for CMOC extraction

We extracted CMOCs from 34 articles that we rated as high for relevance and richness as they provided adequate details for theory development on community engagement in MPDSR [10, 20, 21, 27, 48, 51–53, 60, 61, 65–67]. We also extracted CMOCs from 15 articles that we rated as medium for relevance and richness for theory refinement [11, 24, 26, 29, 54, 62–64, 68–74]. We rated 11 articles as low because they did not provide enough description of community engagement [15, 25, 55–57, 59, 76–80]; and thus excluded these articles from the CMOC extraction as they did not provide adequate information to produce or refine theory. We also identified six studies as sibling texts that we included for CMO extraction, because the articles provided additional details on the context and mechanisms of studies that were rated as either high or medium in richness and relevance [28, 49, 58, 81–83]. See Table 5 for sibling studies.

**Table 5:** Sibling studies

Included study	Sibling studies	intervention
Hutain et al., (2019) [20]	O’Connor et al. (2019) [82]	RCT on community based health information systems
Bakhbakhi et al.; (2017); Burden et al (2021) [60, 61]	Bakhbakhi et al. 2018, 2019 [49, 50]	Parents engagement in perinatal death reviews
Biswas et al. 2014, 2016, 2017, [11, 22, 48]	Biswas et al. (2018) [28]	Community engagement in death notification and reporting Community based reviews (social and verbal autopsy)
Kalter *et al. *(2011) [51]	Dikid et al. *(*2013) [58]	UNICEF supported maternal and perinatal death review programme in India (MAPEDIR)
Melberg et al. (2019) [71]	Melberg *et al. *(2020) [83]	MDSR implementation in Ethiopia

Initially, we extracted 40 CMOCs from the 34 articles included in the review. Upon discussion with co-authors and a steering committee drawn from the WHO technical working group, we merged these into 20 CMOCs. The 20 CMOCs were synthesised and used to refine the initial programme theories developed in the first stage of the realist review process.

This synthesis resulted in 5 programme theories explaining how community engagement in MPDSR works, for whom it works and the different outcomes produced. See Table 6

**Table 6:** Programme theories on community engagement in MPDSR

Programme theory (PT)	CMOCs that contribute to the theory
PT 1: Fear of blame demotivates community members and health workers from engaging in MPDSR	2 CMOCs: 16 and 17
PT 2: Communication and feedback among MPDSR participants and stakeholders	3 CMOCs: 18, 9, 13
PT 3: Social connectedness among community members	2 CMOCs: 1 and 15
PT 4: Financial and non-financial incentives motivate community members and health professionals to engage in MPDSR	9 CMOCs: 2, 3, 4, 5, 7, 8, 11, 12, 20
PT 5: Routinisation and integration of community engagement into existing health systems and community processes	2 CMOCs: 6,19

### Programme theory 1: fear of blame demotivates community members and health workers from engaging in MPDSR. (Additional files, Table 7)

This theory explains that health professionals can be reluctant to include community members in facility-based reviews because they associate this with reduced confidentiality, blame and medico-legal risks [10, 49, 50, 83]. This can demotivate health professionals from participating or affect their willingness to give honest accounts of the circumstances that contribute to maternal/perinatal deaths [20, 21, 29, 52, 68, 71].

In contexts where there is a pervasive culture of blame, community members (e.g. husbands) can associate MPDSR with threats of arrest, making them unwilling to report maternal deaths [68, 71]. Community members can also feel ashamed about deaths arising from socially stigmatising events such as illegal abortions or HIV infections, so do not want to report such deaths [20, 21, 29, 52]. Community members can hesitate to contribute at community death review meetings if the family of the deceased is perceived to be reluctant to discuss details about the death. However, when family members actively participate in the discussions, other community members are more willing to share their ideas during the community death review session [52].

Some authors report that blame can be minimised during community death review meetings by emphasising the blame-free nature of the process during training and reiterating this at the start of each meeting [21]. In Bangladesh, health professionals who facilitate social autopsy sessions control the discussion to ensure that blame is avoided [48]. One health worker explained: “*In a few cases during the holding of an SA, the audience raised blame against the health system or health care providers. We minimised that tactfully and concentrated our talk more about complications*”(48: 7). Paradoxically, the lack of a complaints process for community members can increase the likelihood of litigation and blame culture [83].

### Programme theory 2: communication and feedback among MPDSR participants and stakeholders (Additional files, Table 8)

Creating opportunities for dialogue throughout the MPDSR cycle between community members, health professionals, and other stakeholders in the MPDSR process can allow all participants of the MPDSR process to critically reflect on the issues that contribute to deaths. We posit that dialogue allows participants to exchange ideas by discussing different forms of knowledge held by MPDSR participants (both biomedical and community members’ knowledge based on their experiences before an adverse outcome), which can improve the quality of death reviews and trust in the MPDSR process. We identified four models of communication between health professionals and community members which may either facilitate or limit community engagement in MPDSR.

#### Model 1: phased approach, starting with separate spaces for health workers and community members to discuss deaths, before joint discussions

Some programmes create separate spaces where health professionals and community members review deaths at different locations, which increases the likelihood that people can speak freely during the meetings [21, 49, 52, 61]. This can also reduce power hierarchies, and community members can give feedback about health service provision without fear [21, 49, 52, 61]. We posit that phased approaches leverage on social cohesion within each group i.e., among community members or health professionals. By separating initial discussions, similarities between group members can facilitate open communication and feedback during the review. However, the approach is time-consuming and may be difficult to sustain in high mortality settings [21, 61].

Interventions that create separate but conducive spaces for collaboration rely on trusted community intermediaries such as midwives [61], parent advocates [60], health surveillance assistants [21] and a ‘*key participant*^1^*’* [52] who channel the main points of the review and action points from the community group to the health professionals. For example, a pilot study in Malawi created separate spaces for community members and health professionals to review and propose solutions for maternal deaths (steps 1–3) before the two groups could hold joint discussions at public meetings that were mediated by trusted community intermediaries (steps 4 and 5) [21].

A UK study found that frontline health workers such as midwives can mediate between senior consultants and bereaved families by channelling information on parents’ experiences of care to consultants involved in perinatal death reviews [60]. This study demonstrated that working with parent advocates and midwives as mediators can improve communication between families and health professionals during death reviews and provide bereavement care for families [49, 61], but we did not find any examples of this in low-income countries. We would expect that power differences in LMICs can make it difficult for community members to question health professionals during death review meetings. Although this probably differs between different settings in LMICs, in general, it is likely that such power differences are wider in LMICs than in high income countries. This could explain why, in the studies included in this review, communities in LMICs did not consider frontline health workers as trusted mediators, but rather relied on CHWs to play this role.

#### Model 2: Community representatives channel the findings of facility death review meetings to community members

Two studies from LMICs demonstrated that programmes can rely on trusted community representatives to channel findings from facility based death review meetings to community members [21, 52]. Where community representatives or trusted intermediaries participate in death reviews on behalf of the community, the underlying assumption is that they have the agency to share and discuss community experiences with health professionals who are more powerful than them.

#### Model 3: promoting two-way communication during community death review meetings

Several interventions created opportunities for dialogue between health professionals and community members by promoting two-way communication during community death review meetings [20, 27, 29, 49, 58, 75]. These interventions begin by making information about deaths visible to community members using visual tools such as maps or through public community death review meetings so that they can understand how health professionals use death notification reports [20, 21, 27, 29, 48, 62, 65]. Interventions that create opportunities for community members to visualise information on where deaths have happened, can open up spaces for community members to use their knowledge and critically reflect on the circumstances that led to the deaths, which can motivate community members to provide information and ideas on how future mortality can be prevented [20, 21, 27–29, 51–53, 62, 65].

However, sometimes community death reviews are limited to deaths that occurred in the community but not those in health facilities [10, 28, 68]. Health professionals can perceive that community members lack relevant knowledge to engage in discussions about deaths in health facilities [19, 55]. This can result in the exclusion of community members from the review process if their knowledge is not recognised as valuable [49]. Yet, community members also want feedback about deaths that occurred in health facilities to better understand why the deaths happened. If health professionals do not give feedback about these deaths, community members feel unheard by health professionals, mistrust the health system [10, 68] and are more likely to take legal action against health professionals for perceived negligence [83].

#### Model 4: one-way communication from health workers to communities

In some contexts, health professionals use community meetings to provide health education to community members [10, 20, 48]. When MPDSR programmes use uni-directional communication models for community engagement, community members do not have opportunities to share their experiences or receive feedback from health professionals, limiting community willingness to support MPDSR activities [68]. We posit that community death review processes primarily geared towards providing health education to community members work on the assumption that community members lack knowledge and that community meetings provide opportunities for health professionals to increase community knowledge [10, 20, 48, 58, 64, 68].

### Programme theory 3: social connectedness among community members (Additional files, Table 9)

The involvement of community informants or volunteers^2^ who have strong social connections and routine contact with their communities creates trusted channels of communication between community members and health professionals.

When community informants have strong social bonds and connections with the communities they serve, they are more likely to know households where deaths have occurred, which can support death identification and reporting in contexts where vital registration systems are weak [20–22, 27, 29, 51, 52, 63, 67, 72, 82]. Interventions rely on community informants who have routine contact with families, such as community health volunteers who channel information between community members and the health system for death reporting, review, and response [10, 11, 20, 21, 24, 51, 54, 58, 63, 66, 68, 69, 72, 73, 82, 84].

Conversely, in contexts where community members migrate frequently, such as urban slums, it is not as easy for community informants to identify and report deaths because there are weaker social bonds between the informants and these community members [20, 21, 29]. In addition, when community members do not recognise and respect the people selected to act as community informants, possibly because the community members are not involved in selecting the informants, death notification and reporting can be unsuccessful [63, 64].

### Programme theory 4: financial and non-financial incentives motivate community members and health professionals to engage in MPDSR. (Additional files, Table 10)

When programmes allocate material resources that are adequate and fit for purpose to the health system and community members, this provides extrinsic motivation to health professionals and community members and encourages them to support the MPDSR process. Programmes can also use non-material incentives such as recognition and encouragement to intrinsically motivate both community members and health professionals to engage in MPDSR related activities.

Allocating resources to support the training of community informants is an important first step for community engagement in MPDSR, as community informants need technical and interpersonal skills to perform their roles [20, 21, 27, 51, 54, 62, 66, 67, 73, 74, 82]. In Ethiopia, Ayele et al. [13] found that deploying an adequate number of health extension workers who are responsible for the supervision of community informants improved the quality and timeliness of reporting.

Some interventions have also trained CHWs to conduct verbal autopsies [66, 67, 74]. The underlying mechanism is a recognition that CHWs are trainable, and with supervision, they are capable of conducting verbal autopsy interviews, which can free up health professionals’ time and can increase coverage of death reporting and community death reviews [66, 67, 74].

Some community volunteers are expected to travel within their communities to identify households where deaths have happened [20, 24, 69, 72, 73]. In contexts where the physical terrain is difficult to navigate on foot, providing community informants with tools such as mobile phones or tablets can improve efficiency and timeliness in reporting deaths from the community to the health system, provided an adequate mobile network signal is available [24, 73]. Conversely, notification and reporting of community deaths can be hampered if informants do not receive the necessary logistical support [63]. Community volunteers also require a supportive environment to perform their roles. For instance, the “*arduous nature of CHW work”* led to resignations among CHWs in Pakistan (78:3). In some contexts, programmes use financial incentives to motivate community informants to identify and report deaths [54]. In high mortality-low resource settings, it can be difficult to sustain payment of community informants for death notification and reporting because of the financial implications [67].

Some interventions used non-financial incentives to motivate community engagement in MPDSR [21, 27, 67]. By recognising and valuing community members’ capabilities and assets, programmes communicate that their contributions are useful and valid, which intrinsically motivates them to engage in MPDSR processes. When they are mobilised and motivated to participate in community death reviews, this can encourage a sense of self-efficacy and confidence among community members, and they can use their material resources and capabilities to propose and support implementation of recommendations made during MPDSR [21, 28]. For example, engaging community members in joint problem-solving forums resulted in a community member donating land to construct a health post in their community [70]. Community members also use their critical reflection skills to review and question traditional practices contributing to deaths and propose solutions to address the harmful traditional practices [21, 27, 28, 52].

Programmes that improve community engagement in death notification, reporting and review also increase the workload of frontline health professionals and the senior staff who support them [21, 22, 27, 48]. Allocating adequate financial resources to the health system at different levels can ensure that adequate and trained human resources are available to support community engagement in MPDSR [10, 22, 48, 65]. Providing resources to meet the cost of deployment and training of health professionals can reduce risks of burnout by ensuring that workloads associated with community engagement activities, such as supervision and mentoring of informants, are reasonable [63].

When front-line health professionals are supported by senior health leaders at subnational and national level and are provided with symbolic resources such as encouragement to support community engagement in MPDSR activities, frontline (primary care) health professionals can be motivated [51]. Frontline workers are also more likely to prioritise community engagement activities because they associate community engagement in MPDSR with meanings of value and importance [51].

In many low resource settings, community members and health systems lack material resources to implement solutions identified during the death review processes [21, 27, 28, 51]. When health professionals and community members do not see any changes after making recommendations because there are no resources to support implementation, community members can disengage from the MPDSR process [21, 27, 51].

Community engagement can also be a means through which the health system and community members mobilise material and non-material resources to support MPDSR implementation. We identified two mechanisms that facilitate resource mobilisation. Firstly, community death review meetings provide the opportunity for community members to advocate directly with their leaders and health professionals [20, 21, 28, 51, 62]. Community members can use social pressure to motivate duty-bearers and health professionals to implement action plans made during public meetings [21]. Secondly, community engagement in MPDSR can connect community members to external actors such as NGOs to address social structural barriers that contribute to maternal and perinatal deaths [27, 51, 53, 55, 62]. Community members can leverage on financial resources from civil society organisations (CSOs), private sector or NGOs working in their communities to implement the proposed solutions [27, 51, 62]. Programmes can also use the community engagement process to leverage non-material resources from CSOs, such as giving voice and holding policymakers and health professionals accountable for implementation of recommendations.

### Programme theory 5: routinisation and integration of community engagement into existing health systems and community processes. (Additional files, Table 11)

Interventions that establish routines or build on existing health system structures and processes are more likely to be scaled up or sustained over the long term because they support normalisation of health system functioning and community practices related to MPDSR [26, 27, 69, 72, 73, 80, 85]. Working with existing community informants or volunteers and health system policies for community engagement is more likely to be sustained or scaled up [20, 27, 73, 82].

When community-based health information systems are integrated with sub-national and national health information systems, data that community members collect is more readily available for decision-makers at different levels of the health system to support the implementation of recommendations [20, 22]. In Bangladesh, established routines for death notification and reporting through an integrated health management information system ensures that data reported at primary care facilities can be used for decision making to make changes at local level [22].

When programmes establish calendar routines for submitting death notification reports, e.g. within 24 h of a death, weekly or monthly reporting, they set up rhythms of practice, which normalises the reporting process among community informants and makes it easier to monitor and supervise their performance [10, 20, 22]. If death notification and reporting processes are standardised across the health system, it is possible to avoid duplication of effort as different interventions avoid using different reporting formats and HMIS systems [20, 82]. Interventions which strengthen existing reporting tools are conducive to more efficient training and supervision of community volunteers [20].

If interventions introduce innovative strategies for community engagement in MPDSR, but these are not integrated into the existing health system, it is less likely that the health system will sustain or scale them up. Several articles described pilot studies [20, 21, 27, 52, 54] or programmes supported by NGOs for a limited time [51, 58, 62, 73]. For example, while the intervention in Malawi was well received by health professionals and community members, it was unclear to the authors if the innovative five-step approach for community linked maternal death reviews would be adopted as routine practice [21].

Only studies from Bangladesh described community engagement in MPDSR as part of the health system's routine function [11, 22, 28]. Correspondence with an author of a number of papers from Bangladesh [11, 22, 28] provided additional insights on the mechanisms that have supported routinisation and integration of community engagement in MPDSR. The author noted that integration of community engagement in MPDSR has been facilitated by several factors related to the programme theories we have presented. For instance, routinisation and integration requires material and non-material resources (PT4) such as commitment and encouragement of senior health professionals to frontline health workers who facilitate social autopsy sessions in the community. In addition, the MPDSR programme in Bangladesh benefitted from material resources from external actors (such as the UN system) in the earlier years of its implementation and over time, the external actors have continued to advocate with the national government to allocate resources for community engagement. At the same time, external actors (e.g. UN system) have worked with frontline health workers and community members to promote social autopsy as a learning tool through dialogue (PT2). Frontline health workers are expected to conduct social autopsy as part of their routine community health activities within the catchment areas of the primary care facility. Thus, through simultaneous top-down and bottom-up approach to mobilise material and symbolic resources and community dialogue, community engagement has been prioritised in national budgets to support training and supervision of frontline health workers and community members are motivated to engage in MPDSR without financial incentives for their participation.

## Discussion

This realist review has produced five programme theories explaining the contexts and mechanisms that facilitate or limit community engagement in MPDSR. PT 1 explains that when MPDSR participants and stakeholders associate community engagement with negative meanings of blame, risk, shame, and lack of confidentiality, they do not consider MPDSR sessions to be safe participatory spaces. PT 2 illustrates that creating opportunities for dialogue, where health professionals and community members have discussions and feedback sessions about the findings of death reviews in the community and within health facilities, can improve the quality of death reviews. Programmes can work with trusted mediators and intermediaries, who connect the different participatory spaces by channelling information while maintaining confidentiality and minimising power hierarchies and blame culture. PT3 shows that physical and social proximity among community members enables community informants to build strong social bonds with community members, which facilitates death notification and reporting. PT 4 demonstrates how programmes can use financial and non-financial incentives to motivate health professionals and community members to support surveillance and response. PT 5 illustrates that community engagement in MPDSR is likely to be sustained in contexts where it is routinised and integrated into existing health system processes such as HMIS, health budgets and workplans.

The findings of our review align with other theoretical frameworks on community engagement in health. Renedo and Marston’s [86] theoretical framework on the dimensions of participatory spaces illustrates that community engagement is carried out in spaces that have symbolic and social dimensions. The symbolic dimensions are the meanings and connotations that participants, such as health professionals and community members, associate with the participation process [86]. Our programme theories explain that health professionals and community members can associate community engagement in MPDSR with negative meanings of blame, power hierarchies or shame (PT 1), creating unconducive participatory spaces. Health professionals and community members can also associate MPDSR participation with positive meanings of importance by allocating material and symbolic resources and recognising existing capabilities and assets (PT 4), which creates enabling environments for community engagement.

Our programme theories also mirror other literature on social accountability and the role that community engagement can play in connecting community members with material and symbolic resources from external agents such as NGOs [87, 88]. The engagement process can create spaces for the poor to network with powerful external agents who strengthen community voice to demand change [89, 90]. PT 4 illustrates that programmes can either use community death review meetings to advocate with their leaders directly [21, 51] or connect community members to external agencies who can advocate on behalf of the community [53] or provide the material support that community members need to implement MPDSR recommendations [27].

Creating separate spaces for health professionals and community members during death reviews can minimise power hierarchies and blame culture, which is conducive for collaboration (PT2). This could improve the quality of the information generated at death review meetings as health professionals provide opportunities for community members to share their experiences of care and use this knowledge to make changes [21, 50, 61]. Through dialogue, community members can feel that their knowledge and experiences are recognised and valued by health professionals (PT 3), which can motivate them to allocate material (such as land or finances) and non-material resources (such as time) for implementation of responses [21, 27, 70]. Practically, this could mean that in the initial stages of the death review process, health professionals meet separately from community members, and at a later stage, the two groups meet for a joint discussion. This process would require trusted mediators such as parent advocates or community health workers who channel information between health professionals and community members.

In their analysis of community participation, Morgan [91] has shown that community engagement^3^ can be understood from either an instrumental lens or an empowerment/social transformation lens. Instrumental approaches use the engagement process as a tool for improving efficiency or sustainability of programmes [91]. PT 5 illustrates the instrumental ways in which community engagement in MPDSR uses routinisation and integration to improve efficiency and sustainability of programmes. PT 3 also shows that the MPDSR process relies on existing community relationships and social bonds as an instrumental process for death notification and reporting. Community engagement for purposes of empowerment involves critical reflection, dialogue (PT 2) and providing resources to address the structural barriers that limit social change (PT 4).

### Implications for practice

Communities are not yet widely involved in MPDSR. The critical first step in ensuring that both health professionals and community members are willing to participate in MPDSR related activities is to address fear of blame. Strategies that could address this include ensuring a legal framework that protects health professionals from threats of litigation and training health professionals and community leaders on the objectives of the MPDSR process [92]. Poorly managed community engagement in MPDSR could further exacerbate blame culture, but if well managed, it may also provide opportunities to address blame culture. For instance, health systems could engage community members in developing complaints processes to feedback cases of dissatisfaction with health services or complaints about perceived health worker negligence.

Establishing dialogue requires that both health professionals and community members value and recognise each other's contributions by minimising power hierarchies and blame culture. This could improve the quality of death review meetings and increase the likelihood of community support in the implementation of local-level recommendations, such as addressing harmful traditional practices or contributing material resources to support response efforts [21, 27, 52]. Health professionals can learn about community members’ experiences of care and the circumstances that could have contributed to an adverse outcome [93]. Community members can learn from health professionals about risk factors that contribute to deaths and improve their care-seeking behaviour [21, 70].

While it may be potentially time consuming, setting up separate but collaborative participatory spaces to review deaths and working through trusted mediators could allow community members and health professionals to establish dialogue. In high mortality settings, it may be more useful to sample and only review a representative number of cases using this approach, as it is likely that the modifiable factors are similar in many of the cases under review [29].

Most of the articles that we included in this review described interventions that were either initiated by NGOs or research teams. They were not integrated into the health system and could not be sustained after the funding/research study period, except in Bangladesh. In some instances, interventions were implemented in line with existing routines, such as working with the existing network of community informants for death notification and reporting and using or improving the existing Ministry of Health forms for notification, verbal autopsies and death reviews [20, 21, 27]. However, the failure to integrate the innovations piloted by different research teams and NGOs into the existing health system budgets and workplans meant that the programmes were unsustainable. More research to understand mechanisms that can support the routinisation of community engagement into MPDSR would be useful.

While there are differences between settings how the programme theories work, these theories explain that community engagement in MPDSR is relevant regardless of mortality rates and income levels. The fear of blame and the negative meanings associated with the MPDSR process (PT 1) can influence the willingness of health professionals to collaborate with community members in both high- and low-income countries. Strengthening social connections among community members and promoting dialogue for communication and feedback over monologic communication models (PT 2 and PT3) can improve death reviews in both high- and low-income settings. Both high-income and low-income contexts can rely on trusted mediators to facilitate dialogue and channel information from community members to the health system and from the health system to community members. Health professionals and community members in both high- and low-income countries can be motivated to support community engagement if they feel their contributions to the MPDSR process are recognised and valued (PT4). Finally, innovations such as patient-centred communication models (e.g. sending bereaved families emails or letters to get their inputs on their experiences of care in high-income countries [40]) need to be tested in different contexts before integration into the health system so that these activities can be sustained in the long term.

Our programme theories are data-driven and differ from the initial programme theories we identified in the first stage of the review [30]. Our initial programme theories described how community engagement works in different parts of the MPDSR cycle as discrete activities, for example, only engaging community members in death notification and reporting with no engagement in other parts of the MPDSR cycle. Our refined programme theories show that community engagement in MPDSR is more likely to produce positive outcomes if all five programme theories are seen as part of one whole. The mechanisms that support community engagement should be implemented as a package rather than fragmented and implemented only in some stages of the MPDSR action cycle.

The programme theories could be applicable to community engagement in other maternal and newborn health (MNH) interventions, such as quality of care improvement programmes where collaboration and interaction between health professionals and community members is desirable. By examining the relationships and communication models among participants and the meanings that people associate with the participatory spaces, we can use these programme theories to design other MNH interventions. Similarly, by establishing routines and integrating community engagement into health system functions and budgets, it is more likely that MNH interventions can be sustained and be implemented more efficiently.

### Strengths and limitations of the realist review

To the best of our knowledge, there have been no other studies that have developed or refined theories on how community engagement in MPDSR works and the mechanisms that trigger positive or negative outcomes. We have taken a robust, systematic approach to draw from the wider literature on community engagement in health to refine our IPTs. Through its elicitation of the generative causation of how community engagement works within MPDSR, this review provides explanatory information which can support implementation. Given its focus on triggering mechanisms across varied contexts, this review can also support context-specific implementation and gives a more nuanced understanding than reviews which do not unpack such elements.

Although every effort was made to include all relevant articles, some essential resources may have been missed, especially as the search was conducted in English only. Additionally, the rating of richness and the associated data extraction may have influenced some of the results. However, this process was followed to best manage the quantity of articles and the need for articles with enough explanatory depth [42]. Another shortcoming is the limited number of articles from high-resource contexts, which limits the explanatory power for these settings.

Most of the articles did not explicitly refer to community engagement theory. As well as contacting two authors of included studies, our previous knowledge of relevant health literature and practical experience implementing community engagement in MPDSR (and MNH programmes) also informed our analysis of the mechanisms that trigger outcomes in different contexts. The experience and knowledge base of the co-authors and our advisory committee is a strength, as we have sought to produce practical programme theories to explain why community engagement in MPDSR works or does not work in different contexts.

### Priorities for further research

Fear of blame remains a huge deterrent for community engagement in MPDSR, and it may be useful to use participatory research approaches to explore how blame and other negative meanings associated with MPDSR participation can be managed. It is also important to better understand how the legal and regulatory environment can be improved to reduce the blame culture.

The pilot interventions [21, 49, 61] included in this review demonstrate how community engagement in facility death reviews can be implemented by separating the discussions of health professionals from those of community members. The process described in these pilots is lengthy and may be difficult to sustain in high mortality settings; more research on how to implement MPDSR in separate but collaborative participatory spaces would be useful.

Furthermore, community involvement is more expensive and time-consuming than simply conducting facility-based death reviews. The extra cost and time can be better justified if it significantly improves the effectiveness in terms of reducing mortality and improving quality of care. More research on the cost-effectiveness and financial sustainability of community engagement is needed.

The studies we included in this review did not explicitly explore how issues of power between community members, health professionals and other stakeholders can affect community engagement in MPDSR. A political economic analysis approach would be useful to examine how to minimise power hierarchies in the MPDSR process and how to advocate for resource mobilisation to support response.

## Conclusions

Implementing community engagement in MPDSR requires a systems approach that supports implementation of the five Programme Theories collectively rather than implementing community engagement in specific parts of the MPDSR cycle as our initial programme theories had suggested. Community engagement is useful for implementation of MPDSR both in the community and in health facilities and in both high- and low-income settings.

## Supplementary Information

Supplementary Material 1.

## Data Availability

All data analysed during this realist review are included in this published article (and its supplementary information files).

## Abbreviations

CBHIS: Community-Based Health Information System
CHW: Community Health Worker
CLMDR: Community-Led Maternal Death Reviews
CSO: Civil Society Organization
DHIS: District Health Information System
ENAP: Every Newborn Action Plan
HIC: High Income Country
IPT: Initial Programme Theory
LMIC: Low- and Middle-income country
MNH: Maternal and newborn health
MDSR: Maternal Death Surveillance and Response
MPDSR: Maternal and Perinatal Death Surveillance and Response
PT: Programme Theory
SDG: Sustainable Development Goal
TWG: Technical Working Group
WHO: World Health Organisation

## Footnotes

1 Key participant described as a ‘knowledgeable community member who has a higher social status than other community members and is known and respected by the community.

2 Different articles refer to community members who support MPDSR as either community informants, volunteers, or community groups. For consistency, we will use the term community informants to refer to community members who are engaged in MPDSR activities.

3 We use the term community engagement interchangeably with community participation.
