# Supplementary material for: Community engagement in maternal and perinatal death surveillance and response: a realist review
Source: BMC Pregnancy Childbirth. 2025 Oct 14;25(Suppl 1):1086. doi: 10.1186/s12884-025-08183-x (PMC12522978; doi:10.1186/s12884-025-08183-x)
Supplement: Supplementary file 1 — Supplementary Material 1. [file 12884_2025_8183_MOESM1_ESM.docx]

**Additional files (tables 7-11) CMOCs contributing to programme theories**

**Table 7**

| PT 1: Fear of blame demotivates community members and health workers from engaging in MPDSR. | | | | |
| --- | --- | --- | --- | --- |
| **CMOC** | **Context** | **Mechanism** | **Outcome** | **Ref** |
| 16 | Blame culture is prevalent; threats of litigation for health professionals. | Health professionals associate MPDSR sessions with meanings of risk because of reduced confidentiality in the review process. | Health professionals are unwilling for community members to attend review sessions. | (10,64,65,73,77,83) |
|  | High emphasis on reducing maternal deaths with no mechanisms for minimising blame for health professionals and community members. | Community members associate death notification and reporting with risk of arrest.  Health professionals associate MPDSR sessions with meanings of legal and professional risk. | Reduce community willingness to report deaths.  Health professionals are evasive when participating in death review meetings. | (10,73,77,83) |
|  | No complaints process for perceived negligence of health professionals. | Community members are not aware of how to channel their complaints about deaths.  No clear communication between the health system and community members for perceived negligence. | Increased likelihood of litigation against health professionals and blame culture. | (10,83) |
| 17 | Some deaths are socially stigmatised. | Community members have negative social meanings about these deaths and can associate engagement in death reviews with feelings of shame or blame for deaths. | Stigmatised deaths are difficult to identify, report or discuss in community-based reviews. | (21,27,29,54,66) |

Table 7: CMOCs contributing to PT 1

**Table 8**

| Programme theory 2: Communication and feedback among MPDSR participants and stakeholders. | | | | |
| --- | --- | --- | --- | --- |
| **CMOC** | **Context** | **Mechanism** | **Outcome** | **Refs** |
| 18 | Separate but collaborative spaces for community members and health professionals to review anonymised death reports at separate events and then share their findings at joint sessions. | Increase confidentiality in the review process by ensuring that each group (professionals or community members) only access relevant information that is necessary for the review process.  Creates conducive environments for dialogue and feedback by minimising power hierarchies and blame culture between health professionals and community members. | increased the quantity of information available to guide the review process,  Potentially more honest and open discussions about circumstances contributing to deaths.  Boost health professionals’ morale and willingness to engage community members in the review process. | (21,54,63,64) |
|  | Health professionals are flexible in communicating with bereaved families and community members. | Work with trusted mediators who have ongoing relationships with pregnant women and their families, e.g., midwives and CHWs who channel feedback from health workers to the community and vice versa.  Work with communities to establish patient-centred 2- way modes of communication, e.g., home visits in LMICs or letters and phone calls in HICs. | Build trust between community members, bereaved families, and health professionals.  Increase cooperation between health professionals and community members in supporting the implementation of recommendations.  Empower community members to propose creative solutions and monitor progress in implementing recommendations in the community and within the health system.  Lengthy process that may be difficult to sustain in high mortality settings. | (21,54,63,64) |
| 10 | Frontline health professionals use community death review sessions to make information about deaths visible to community members by facilitating discussions among community members for deaths happening within health facilities and in the community.  Frontline health workers invite community representatives or trusted mediators to facility death reviews to make information about deaths visible to the representatives and holding discussions about community members’ experiences of care. | Community leaders understand how health professionals use the death notification information that community informants report.  Two-way communication between health professionals and community members is established.  Recognition and value of community members’ capabilities and assets, such as local knowledge, which boosts community confidence to engage with health professionals.  Trusted community representatives who have the agency to share community experiences and inputs during death review meetings at health facilities. | Community engagement in death notification and reporting is likely to be sustained.  Improve the quality of death reviews in the community and within health facilities.  Community members can propose solutions and mobilise material assets within the community to support the implementation of responses at the community level.  Improve health-seeking behaviour among community members and address social practices that contribute to deaths.  Better bereavement care for families. | (21,22,27,53,54) |
| 13 | Death review meetings are led by senior health professionals and consultants, ensuring the participation of frontline/primary care health professionals who work directly with pregnant women, such as midwives, to participate on behalf of community members. | Rely on trusted mediators who have ongoing relationships with pregnant women and their families to channel information about patients’ experiences of care before an adverse outcome. The trusted mediators also give feedback about the findings of the review session from consultants to families. | Can improve feedback to family members on review findings.  Provide better bereavement care, which can increase trust in the MPDSR process.  Improve the quality of death reviews. | (63,64) |
| 9 | Frontline health workers use community death review sessions to make information about deaths visible to community members by “educating” community members without giving them opportunities to share their experiences with health professionals. | Uni-directional 1-way communication model where only the knowledge and experiences of health professionals is used.  Fails to recognise and value community knowledge or experiences which demotivates community members. | Community members feel unheard by health professionals and do not have opportunity to give health professionals feedback about their dissatisfaction with health services.  Community informants are not motivated to continue in death reporting or implementing recommendations. | (10,22,48,53,60,77,86) |
|  | Community members are engaged in death notification and reporting, but there is no form of community death review, and community members do not receive feedback about deaths happening in health facilities. | No communication or discussion between health professionals and community members.  Community members’ questions about deaths both in the community and within health facilities are not responded to | Community members are unwilling to identify and report deaths to the health system.  Community members feel unheard and mistrust the health system.  Increased likelihood of legal action against health professionals. | (10,22,48,53,60,77,86) |

Table 8: CMOCs contributing to PT 2

**Table 9**

| **PT 3: Social connectedness among community members.** | | | | |
| --- | --- | --- | --- | --- |
| **CMOC** | **Context** | **Mechanism** | **Outcome** | **Refs** |
| 1 | Majority of deaths are in the community, and weak vital registration systems  Selecting community informants such as priests, shopkeepers/drug sellers, TBAs, and CHWs | community informants have routine contact with families, time, and local knowledge of community life. | shifts death surveillance from a passive process to more active forms that are efficient and cost-effective compared to relying on demographic health surveys or other surveillance systems. | (10,11,20,21,24,53,56,60,67,70,74,75,77,78,82,84) |
| 15 | Vital registration systems are weak, and a proportion of births and deaths happen in the community. there is an existing network of community informants who are trusted and respected in the community, e.g. teachers, religious leaders, traditional birth attendants, and existing community health workers. | Social connectedness between bereaved families and community informants enables them to identify and report deaths to the health system. | Community willingness to engage in MPDSR processes, more complete death reporting and participation in review meetings.  Community informants can also provide bereavement support, which strengthens socia**l** bonds. | (20–22,27,29,53,54,67,71,74,82) |
|  | Community informants are not recognised and respected; they may not know families well and do not have strong social bonds (where populations are mobile, e.g. in urban slums) | Community informant roles are not valued, and they are less connected to the community. | Community members are unwilling to provide information to the informants.  under-reporting of deaths. | (20,21,67,68) |

Table 9: CMOCs contributing to PT 3

**Table 10**

| **PT 4: Financial and non-financial incentives motivate community members and health professionals to engage in MPDSR** | | | | |
| --- | --- | --- | --- | --- |
| **CMOC** | **Context** | **Mechanism** | **Outcome** | **Refs** |
| 2 | Majority of deaths happen in the community. | Allocating resources for training community informants and their leaders equips community volunteers with the necessary interpersonal and technical skills, which builds the capacity of the informants and community leaders. | Increase likelihood that community informants will complete notification forms accurately.  Increase likelihood that community leaders will support community informants to perform their roles. | (20,21,27,53,56,59,66,70,71,82) |
|  | Majority of deaths happen in the community, and community informants are engaged in death notification and reporting, but submission of reports is prioritised without a supportive environment. | The lack of resources for training community informants means that informants do not have the necessary technical skills to complete forms accurately.  Lack of resources to support supervision demotivates health professionals from supporting community informants as they perform their roles. | Notification reports of poor quality (e.g., missing data). | (20,56,82) |
| 4 | Health system ensures that adequate resources (time and money) to support community volunteers are allocated by paying an adequate number of extension workers to supervise and monitor community informants. | Health workers feel that notification and reporting of deaths is recognised as a priority for the health system.  Community volunteers/informants feel that the health system values and recognises their work as useful. | Improvement in quality and timeliness of death notification and reporting.  Better monitoring of community informants.  Better linkages with households. | (10,20,21,24,53,70,75,78,82) |
|  | National and subnational health system levels want to engage community members, but no material resources are provided to primary care facilities to support community volunteers in their notification and reporting duties. | Failure to provide material resources to support community engagement activities communicates that health system leadership does not prioritise community engagement.  Community informants are demotivated. | Community informants are not adequately supported and supervised to perform their roles.  Notification reports are of poor quality, and deaths are underreported.  Difficulties sustaining community engagement in death identification and reporting. | (67,78) |
| 5 | Resources are provided for training and logistical support for community informants to report deaths, such as providing a transport allowance or giving mobile phones, smart tablets, or airtime to community informants. | Makes it easier for community informants to relay death notification reports to the health system.  Motivates community informants working in difficult contexts to sustain their support of death notification and reporting. | Improve timeliness and efficiency of notification and submission of death reports. | (11,24,67,75,78) |
| 12 | Health system wants to increase community engagement in death notification, reporting, and review.  Senior health leaders at subnational and national levels provide non-financial support, such as encouragement for community engagement in MPDSR activities. | Providing adequate resources for implementing recommendations, such as addressing quality of care issues identified through the review process, communicates to health workers and community members that the MPDSR process is useful for initiating change.  Frontline health professionals associate community engagement with meanings of value. | Increase in number of deaths reported.  Improve the morale of health workers to review deaths in health facilities and in the community.  Motivate community members to make and implement local-level actions to prevent future deaths and improve health-seeking behaviour.  Frontline health workers are more likely to prioritise community engagement in MPDSR. | (21,27,28,53) |
| 11 | Responsive health system or duty bearers that support the implementation of recommendations made by the community. | Providing resources to community members to implement recommendations communicates trust in the community’s ability to solve problems. | Implementing community action plans to address modifiable factors that they identify in the community and within the health system.  Increase community motivation, confidence, and self-efficacy. | (20–22,27,48,53,54,60) |
| 3 | Community informants engaged in death notification and reporting, but reports are of poor quality and need improving. | Training community informants, paying them for each accurate death identified, monitoring and reviewing each notification report.  uses financial incentives to motivate community informants to report accurately. | improve the quality of death notification data by avoiding double counting and increasing coverage. | (56) |
| 7 | Community informants collect information on deaths and submit reports to the health system without financial incentives, and health professionals involve community members in community death reviews. | Community members feel that health workers value the information collected and they are motivated to support death notification, reporting, and review. | Sustained levels of reporting and community death reviews despite the lack of financial incentives. | (21,27,71) |
| 8 | High number of community deaths and an existing network of CHWs, but low levels of community-based death reviews. | Training CHWs in interpersonal skills, bereavement care and verbal autopsy interviewing skills can build the capacity of CHWs and shift tasks from health workers to CHWs to conduct verbal autopsy.  Task shifting of verbal autopsy interviewing roles from health workers to CHWs | More deaths are reviewed.  Bereavement care is provided to families. | (71,76) |
| 11 | Community members are engaged in review processes, but recommendations are not implemented. | Loss of faith in the engagement process.  False hope for both health professionals and community members. | Unwillingness of community members to identify and report deaths. | (53,60) |
| 20 | Senior health providers and leaders are not supportive of death review processes held in health facilities. | The review process is not prioritised and is viewed negatively (e.g., as time-consuming). | Frontline health workers are not motivated to give feedback to community members about the findings of death review meetings. | (10,77) |
| 14 | Public health system is weak; MPDSR relies on financial resources provided by external actors such as NGOs or private sector. | Leverage on financial resources from  external agents such as NGOs to support implementation of MPDSR activities.  Leverage on non-financial (symbolic) resources from external actors such as NGOs and civil society to advocate on behalf of the community or support social accountability activities that empower communities to voice their demands to duty bearers. | Health system can use the resources provided by external actors to to support implementation of MPDSR related activities.  Community members can hold health professionals and duty bearers accountable for the implementation of MPDSR recommendations. | (20,21,27,48,53,60,82) |

Table 10: CMOCs contributing to PT 4

**Table 11**

| **PT 5: Routinisation and integration of community engagement into existing health systems and community processes.** | | | | |
| --- | --- | --- | --- | --- |
| **CMOC** | **Context** | **Mechanism** | **Outcome** | **Refs** |
| 6 | Routine data collection for vital events exists but is weak. | Training, continuous monitoring (through regular meetings and phone calls) and supervising community informants to use simplified data collection tools such as logbooks or village registers to report deaths can strengthen community-based health information systems (CBHIS). | Community death notification is routinised which can improve the efficiency of reporting deaths | (20,22,53,68,71,74,77,82) |
|  | CBHIS data is integrated into the existing national health management system. | Improves efficiency in uploading data into sub-national and national health management information systems such as DHIS. | Data on maternal and perinatal deaths is readily available to guide decision-making at different levels of the health system. | (11,20,22,28,48,52,82,84,86) |
|  | Notification reports from the community are not systematically linked to existing health information management systems. | Information on deaths reported by community members cannot be accessed in a systematic way | it is more likely that deaths reported by community members will not be reviewed. | (68). |
|  | the MPDSR process is integrated into existing health system processes for community engagement, e.g. integrated into existing health system budgets and working within the existing community engagement strategies set by the health system for training and supervision of community informants/volunteers. | leverages on the existing health system resources and avoids duplication of effort | CE in MPDSR activities is likely to be sustained | (11,22,28,48,52,84,86) |
| 19 | Community engagement activities or innovative strategies supported by external actors such as NGOs are not integrated into the health system plans and budgets. | Health system does not allocate resources (material and non-material) to support continuation of innovative activities. | MPDSR activities will stop when the programmes implemented by external actors come to an end. | (20,21,27,53,54,60,63,82) |

Table 11: CMOCs contributing to PT 5
